# 

**Published:** 1861-03

**Authors:** 


### RECEIPTS NOT HERETOFORE ACKNOWLEDGED.

C. A. Butcher, R H Green, E P Tabbott, Z J McMasters, D M Cool, J J Reed, Jas Brown, J H Everett, J A Ward, H P Allen, John Loftin, J C Michener, J T Keables, P H Ellsworth, John Guffin, B Ward, E W Keegan, W C Thompson, A Garwood, J L Magee, D Dunlavry, C F Little, B Wilson, C S Tucker, J R Webster, Dr Kittle, R W Pierce, B A Bradford, E H Winston, D Kirkpatrick, Jones & Butler, J M Steele, A P Adams, F P Mauley, J S Brengle, H M Lilly, J J M Augear, Jas Thompson, J B Corey, R M Crowder, G W Albin, J H Orr—All of the above $2.00 each for vol. 4. S C Gray, vol 3, $2; M H Bonnell, vols 3 and 4, $4; J N Allen, vol 3, $2; W H Rice, half vol 3, $1; L H Birney, half vol 3, $1: J W Spalding, vols 2, 3 and 4, $6; Lance Samuel, vol 3, $2; J S Skinner, half vol 3, $1; W M Goodwin, vols 1, 2, 3 and 4, $8; B T Buckley, vol 3, $2; S O Long, vol 4, $2: N Teal, vol 4, $2.

